# The ameliorative effects of exogenous inoculation of *Piriformospora indica* on molecular, biochemical and physiological parameters of *Artemisia annua* L. under arsenic stress condition

**DOI:** 10.1016/j.ecoenv.2020.111202

**Published:** 2020-12-15

**Authors:** Saeed-ur- Rahman, Muhammad Khalid, Sadaf-Ilyas Kayani, Kexuan Tang

**Affiliations:** a Joint International Research Laboratory of Metabolic & Developmental Sciences, Key Laboratory of Urban Agriculture (South) Ministry of Agriculture, Plant Biotechnology Research Center, Fudan-SJTU-Nottingham Plant Biotechnology R&D Center, School of Agriculture and Biology, Shanghai Jiao Tong University, Shanghai, 200240, China; b Key Laboratory of Urban Agriculture, School of Agriculture and Biology, Shanghai Jiao Tong University, Shanghai, China

**Keywords:** Arsenic toxicity, *P. indica*, *Artemisia annua*, Transcripts, Flavonoids, Artemisinin

## Abstract

Aim of the current study was to investigate the effect of exogenously inoculated root endophytic fungus, *Piriformospora indica*, on molecular, biochemical, morphological and physiological parameters of *Artemisia annua* L. treated with different concentrations (0, 50, 100 and 150 μmol/L) of arsenic (As) stress. As was significantly accumulated in the roots than shoots of *P*. *indica*-inoculated plants. As accumulation and immobilization in the roots is directly associated with the successful fungal colonization that restricts most of As as compared to the aerial parts. A total of 4.1, 11.2 and 25.6 mg/kg dry weight of As was accumulated in the roots of inoculated plants supplemented with 50, 100 and 150 μmol/L of As, respectively as shown by atomic absorption spectroscopy. *P*. *indica* showed significant tolerance *in vitro* to As toxicity even at high concentration. Furthermore, flavonoids, artemisinin and overall biomass were significantly increased in inoculated-stressed plants. Superoxide dismutase and peroxidase activities were increased 1.6 and 1.2 fold, respectively under 150 μmol/L stress in *P*. *indica*-colonized plants. Similar trend was followed by ascorbate peroxidase, catalase and glutathione reductase. Like that, phenolic acid and phenolic compounds showed a significant increase in colonized plants as compared to their respective control/un-colonize stressed plants. The real-time PCR revealed that transcriptional levels of artemisinin biosynthesis genes, isoprenoids, terpenes, flavonoids biosynthetic pathway genes and signal molecules were prominently enhanced in inoculated stressed plants than un-inoculated stressed plants.

## Highlights

• The level of artemisinin and flavonoids was increased by *P. indica* under arsenic stress condition.
• *P. indica* accumulated and restricted arsenic in the roots.
• *P. indica* showed higher tolerance to arsenic *in vitro.*
• Antioxidant defense system and enzymatic activities were enhanced by *P. indica.*

## 1 Introduction

Being a class one human toxin and carcinogen (Khairul et al., 2017), arsenic (As) is among the metalloids mainly released into the environment by a number of anthropogenic as well as natural activities, particularly in the developing countries (Yaghoubian et al., 2019). Some metalloids, including As, are not essential for biological processes but are toxic even at low concentrations, while others are necessary and effective for metabolic processes (Nongmaithem et al., 2016). As is not only the main causative agent of skin, lung and bladder cancer but also induces a number of illnesses of thyroid gland, cardiovascular and nervous system (Bhowmick et al., 2018). Additionally, the element has been confirmed in 150 minerals so far which contaminate drinking water and ultimately becomes the part of the food chain (Kumar et al., 2015; Sharma et al., 2015). As can be found in both inorganic (arsenate (As (V)) and arsenite (As (III)) and organic i.e. monomethylarsenic acid, dimethylarsenic acid and trimethylarsine oxide forms, the former being most toxic to most life forms while the latter has been recognized as less toxic to living organisms (Francesconi, 2010; Templeton and Fujishiro, 2017; Zhang et al., 2019). Furthermore, arsenate is usually bounded to soil Al- and Fe-containing minerals in aerobic sediments and soils (oxidizing conditions). However, once incorporated into the living system or regardless living system, arsenate reduces into arsenite (most toxic form) and may increase exponentially (Dixit and Hering, 2003; Nisticò et al., 2018). Since a huge number of people suffer from As contamination (Carbonell-Barrachina et al., 2009), the removal and/or reduction of As from polluted soils/waters and its immobilization to avoid bioavailability, is the main concern and have been attracted the attention of researchers recently around the globe (Robberecht et al., 2002). Likewise, As is highly toxic for plant life as it affect the plant water status, reduces plant growth, generate oxidative stress, alter the hormonal content and inhibits photosynthesis, leading to various disorders (especially physiological) and eventually to the plant death (Farooq et al., 2016). In addition, chemically arsenate is phosphate analog mainly transported (mostly in phosphate deficient conditions) via phosphate transporters, thereby damaging vital molecules including DNA and RNA by replacing phosphate in the target molecules. Generally, it is however assumed that most if not all of the absorbed arsenate is reduced to arsenite (by non-specific reductases) in the plant tissues (Tripathi et al., 2012). Furthermore, arsenite can be transported via specific channel (nodulin 26-like intrinsic protein aquaporine channel) and tend to bind to sulfhydryl groups and alters the structures and functions of some proteins (Zhao et al., 2010). Nevertheless, sulfhydryl groups of phytochelatins can bind to arsenite, detoxifying and sequestering it in the vacuoles (Hettick et al., 2015). Plant responses to metal toxicity vary from species to species depending mainly on morphological and phenological stage/development. However, some plants restrict and retain As in their roots (As non-hyperaccumulating plants) while several unicellular organisms, such as, microalgae can resist the detrimental effects of As (Wang et al., 2017) indicating that morphological variation is not pre-requisite for As resistance.

Root endophytic fungus of the order Sebacinales, *Piriformospora indica*, can colonizes the roots of a number of plant species, not only confers resistance against various stresses (biotic and abiotic) (Varma et al., 2013), but also promote the uptake of nutrients (Gosal et al., 2010). In addition, antioxidant defense system of the plant can be enhanced by the fungus which has a vital role in stress tolerance (Vadassery et al., 2009). It has also been shown that the signaling and production of plant hormones such as salicylic acid, ethylene, abscisic acid, gibberellic acid and jasmonic acid can be altered by *P*. *indica* (Peskan-Berghöfer et al., 2015) while the resultant hormonal changes significantly mediate abiotic stress responses (Gill et al., 2016). Several studies have been shown that plant defensive compounds (secondary metabolites) such as forskolin (Das et al., 2014), artemisinin (Arora et al., 2016), curcumin (volatile oil) (Bajaj et al., 2014), bacoside and anticancer podophyllotoxins (lignans) can be enhanced by fungal colonization. The successful colonization and beneficial effects of *P*. *indica* have been confirmed in a number of plant species including Arabidopsis (Siddhanta et al., 2017). Moreover, studies on *Helianthus annuus* (Shahabivand et al., 2017) and *Nicotiana tabacum* (Hui et al., 2015) etc. have also been revealed that *P*. *indica* accumulate different heavy metals, for example, Cd in the roots and restrict their movement to aerial parts. However, response/tolerance of *P*. *indica* itself (*in vitro*) to As stress and the mechanistic explanation of the fungus mediated As stress tolerance and its effects on important secondary metabolite contents (especially, artemisinin and flavonoids) and their biosynthetic pathway genes of *Artemisia annua* L. is not well documented. In addition, several studies, for example (Aftab et al., 2011; Akula and Ravishankar, 2011; Paul and Shakya, 2013; Kumari et al., 2017), have been reported that secondary metabolites (artemisinin) can be increased (up to some extent) under specific concentrations of different heavy metals. Therefore, this study aimed to determine the influence of As stress on growth of *P*. *indica* and synergistic effects of *P*. *indica* and As stress on physiological, biochemical and molecular (especially artemisinin and flavonoid biosynthetic pathway genes) characteristics of *A*. *annua* plants inoculated with or without *P*. *indica*.

## 2 Materials and methods

Assessment of *P*. *indica* for As stress (*In vitro*).

The fungus was inoculated in Erlenmeyer flasks (100 ml) having modified liquid Kafer medium (25 ml) supplemented with different concentrations i.e. 0 (control), 0.01, 0.05, 0.1, 0.2, 0.3, 0.6 and 0.9 μmol/L of As. A 4-mm (approx.) fungal plug, separated from already cultured fungus agar disc, was added to the flasks. The flasks were kept for 15 days in a rotatory shaker at 28 °C at 120 rpm. After 15 days, fungus mycelia were harvested by filtering the culture through Whatman filter paper. Fresh weight of mycelia was measured immediately and oven-dried (65 °C for 24 h) to determine dry weight. Likewise, Hill and Kafer solid agar medium with above stated As concentrations was used for this experiment and fungal tolerance was determined in terms of mycelial growth (circularly extending from the center towards edges). The plates were kept in dark for 20 days at 28 °C in incubator. Fungal growth was determined by measuring the radius of growing hyphae from the center towards the edges of the plates on 5th, 10th, 15th and 20th days after fungal inoculation.

### 2.1 Plant material and growth conditions

Seeds of *A*. *annua* were surface sterilized with sodium hypochlorite (0.1% v/v) for 10 min, and then washed thoroughly (4–6 times) with double distilled water. After that, seeds were grown on the substrate (vermiculite, peat moss and perlite with a 6:3:1 ratio) with a light/dark photoperiod of 16:8 h at 25 ± 2 °C. 25 days old seedlings were transferred to plastic pots containing substrate one week before experimentally contaminated with different As (Na_2_HAsO_4_.7H_2_O) concentrations i.e. 0 (control), 50, 100 and 150 μmol/L with or without *P*. *indica*. Plants were harvested after 30 days; fresh samples were used for some experiments while others were kept at -80 °C for further experimentation. All the treatments consist of 3 biological replicates.

### 2.2 *A*. *annua* and *P*. *indica* co-cultivation

*Periformospora indica* (CBS 125645), obtained from Fungal Biodiversity Centre, Institute of the Royal Netherland Academy of Arts and Sciences (KNAW), was propagated on petri dishes with modified Kafer (Hill and Kafer, 2001) medium at 25 °C in growth chamber. Fungus was further sub-cultured in Erlenmeyer flasks (500 ml) in liquid modified Kaefer medium for 15 days at room temperature at 50 rpm. Approximately, 10 mm mycelium plugs were kept near the roots (1 cm) at the time of sowing.

### 2.3 Root colonization assay

The typhan blue kit (Sangon Biotech Shanghai Co., Ltd.) was used with minor modifications (Phillips and Hayman, 1970; Dickson and Smith, 1998) for the analysis of fungal spores in the roots of inoculated plants. Briefly, root samples were washed thoroughly with tap water and cut into different pieces. Slides were prepared and observed with different magnifications under light microscope connected with a camera. Photographs were taken of the roots successfully colonized with *P*. *indica*.

### 2.4 Assessment of osmotic stress response under As stress

Fresh leaves were used for electrolyte leakage measurement (Lutts et al., 1996). The samples were washed 3–4 times with water and placed in vials having deionized water. After incubation (25 °C for 24 h), the electrical conductivity (L_t_) was determined while the last electrical conductivity (L_0_) was measured after autoclaving the leaves for 20 min at 120 °C.

### 2.5 Electrolyte leakage (%) = (L_t_ ÷ L_0_) × 100

LRWC was determined as described earlier (Smart and Bingham, 1974). Fresh mass (FM) was immediately weighted after collecting leaves from the plants. Leaves were placed in petri dishes having ddH_2_O to obtain turgid mass (TM). Leaves were again weighed after imbibition period, oven dried (85 °C overnight) and reweighed for dry mass (DM) determination. Obtained values (FM, TM and DM) were used using the formula given in Eq. (1) to calculate LRWC.

(1) LRWC(%)=FM−DM÷TM−DM

### 2.6 Determination of As concentrations in root and shoot

For As determination, dry roots and leaves (0.2 g) were used. Plant materials (both treated and control) were digested in HCLO_4_/HNO_3_ (1:4, v/v). After that, the obtained mixture was further extracted using 5 ml HNO_3_ and adjusted to determine volume (250 ml) of ddH_2_O. As concentrations in root and shoot were analyzed using inductively coupled plasma spectrometry (ICP, Thermo Fisher, ICAP7600, USA).

Antioxidant enzyme assays and estimation of proline, MDA and H_2_O_2_ content.

The activities of different antioxidant enzymes were assayed using the kits (Nanjing Jiancheng Biotechnology Institute) spectrophotometrically. The activities (free radical scavenging) by superoxide dismutase (SOD), catalase (CAT) and peroxidase (POD) were determined spectrophotometrically at 550, 405 and 420 nm, respectively. Similarly, the activities of glutathione reductase (GR) and ascorbate peroxidase (APX) were also assayed spectrophotometrically at 290 and 340 nm, respectively. Proline content was estimated in the leaf tissues by spectrophotometric analysis after ninhydrin reaction (under acidic condition) at 520 nm (Jogawat et al., 2013). Malondialdehyde (MDA) was extracted and measured following Thioarituric acid (TBA) protocol (Bao et al., 2009) while H_2_O_2_ content was measured as described previously (Junglee et al., 2014).

Determination of phenolic acid, phenolic compound and flavonoid content.

Arnov method was used for estimation of total phenolic acid (Szaufer-Hajdrych, 2004). The sample (1 ml) was thoroughly mixed with 1 ml of Arnov reagent (sodium nitrite and sodium molybdate each 10 g dissolved in 100 ml of ddH_2_O), 1 ml 0.5 ml HCl, 1 ml 1 M NaOH and 5 ml of ddH_2_O. Finally, ddH_2_O was added to a final volume of 10 ml. Absorbance was measured at 490 nm. Folin-Ciocalteau reagent was used for determination of total phenolics (Singleton et al., 1999). Absorbance was measured at 725 nm. Total flavonoid was determined as described by Lamaison and Carnet (1990). Absorbance for flavonoid content was measured at 430 nm. Flavonoid content and phenolic acid were expressed as quercetin equivalent and caffeic acid μg/g fresh weight, respectively, while total phenolics as gallic acid equivalent in mg/g of fresh weight.

### 2.7 Molecular analysis

TIANGEN, RNAprep pure plant kit was used for total RNA extraction from both control and treated plant samples. RNA quantity and quality was checked using Nanodrop spectrophotometer (Thermo Fisher Scientific Inc., Wilmington, DE, USA) and agarose gel electrophoresis, respectively. Complementary DNA (cDNA) libraries were constructed through prime script RT reagent kit (Takara). Genes expression level was checked by q-PCR analysis and actin was used as internal control. q-PCR data was analyzed using 2^-ΔΔCT^ method.

### 2.8 Artemisinin extraction and HPLC analysis

Leaves were collected from lateral branches and oven dried at 50 °C for 72 h. Artemisinin was extracted from 0.1 g leaf powder using an adjusted ultrasonic processor for 30 min at 30 °C at 55 Hz frequency (Kayani et al., 2019). The resulting samples were centrifuged for 10 min at 12,000 g. Finally, supernatants were filtered through 0.25-μm filters. HPLC analysis was carried out following the method described elsewhere (Lu et al., 2013).

### 2.9 HPLC analysis of flavonoids content

Polyphenols were extracted and measured from both treated and untreated samples with high-performance liquid chromatography (HPLC) following earlier described method (Złotek et al., 2014). Fresh leaves were macerated (25 °C for 20 min) with acidified methanol and centrifuged for 30 min at 9000 g. After that, the collected supernatant was evaporated at 40 °C till dryness. Final volume (10 ml) was obtained by adding ethanol (100%) and extract was prepared. HPLC was performed by following earlier described method (Guo et al., 2005). Different flavonoids such as, syringic acid, ferulic acid, luteolin, chlorogenic acid, quercetin, rutin trihydrate, kaempferol, hydroxycinnamic acids and gallic acid were used as standards.

### 2.10 Statistical analysis

The data were statistically analyzed by one-way analysis of variance (ANOVA), following Duncan's multiple range (DMR) test (SPSS Inc., Chicago, IL, USA). At least 3 replicates were used for each sample, mean and ±SD were also calculated. Significant differences (*P* < 0.05) were calculated.

## 3 Results

### 3.1 Fungal tolerance to As stress (*in vitro*)

**Fig. 1:**
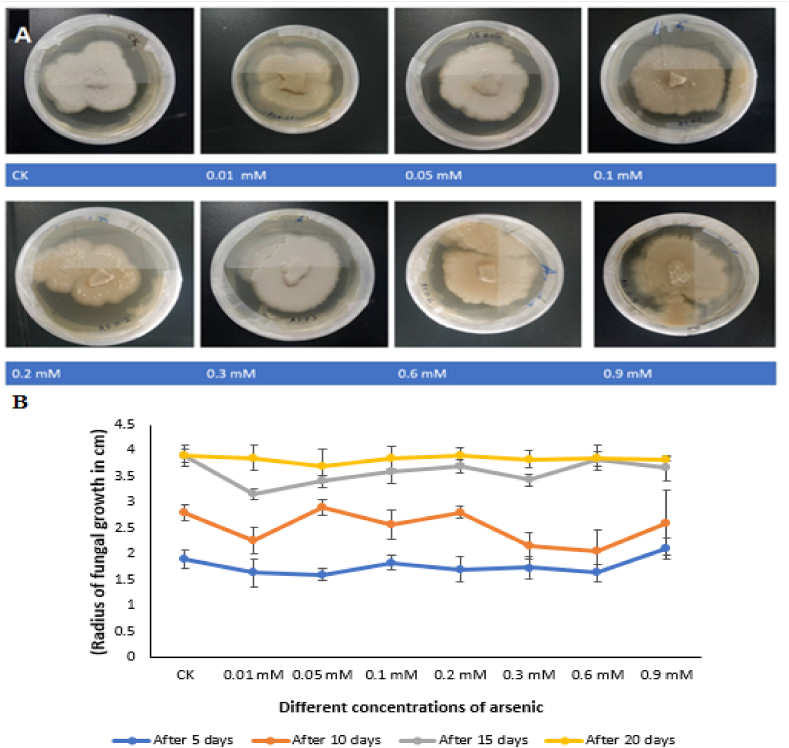
Effect of different concentrations of arsenic on the growth of *P*. *indica* (A–B) *in vitro*. **(A)***P*. *indicia* growth (after 5, 10, 15 and 20 days of incubation) in terms of hyphal extension from the center towards plate edges under different arsenic concentrations (0 mM, 0.01 mM, 0.05 mM, 0.1 mM, 0.2 mM, 0.3 mM, 0.6 mM, and 0.9 mM), (B) Analysis of the radius of fungal hyphae after 5, 10, 15 and 20 days of inoculation. The data is the mean values of three biological replicates with ±standard error.

*P*. *indica* revealed high tolerance to different concentrations of As *in vitro*. The fungus was capable of surviving at higher As concentrations up to 0.9 mM. However, fungal As-toxicity tolerance varied in terms of biomass production. For example, maximum biomass production was shown by fungus at 0.3 mM (in liquid medium assay), even the biomass was higher (25.3 gm) than that of control (20.5 g) (Fig. S1, A-B). However, biomass production was dramatically decreased when the As concentration increased to 0.9 mM, where reduction was observed as 28.9% as compared to control. By contrast, different growth pattern was showed by fungus to the same concentrations of As on solid agar medium. For instance, complete hyphal growth (3.9 ± 0.1 cm) was achieved by control within 15 days while 0.1 mM acquired 20 days to achieve full growth (3.8 ± 0.2 cm) (Fig. 1, A-B). Likewise, 0.1 mM the full growth was achieved by the fungus of the rest of the plates (with different As concentrations) in 20 days except 0.05 mM where full growth was not achieved even in 20 days.

### 3.2 Symbiotic development/*P*. *indica* root colonization

**Fig. 2:**
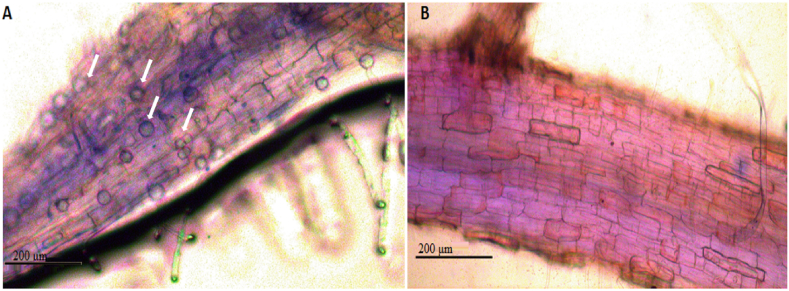
*P*. *indica* colonization in the roots of Artemisia plants (A) Fungal spores inside the roots, spores are indicated with arrows (B) Control. Plants were inoculated with or without *P*. *indica* and supplemented with or without different arsenic concentrations.

Root samples of both inoculated and un-inoculated plants were analyzed for successful root's colonization using staining and microscopy techniques. *P*. *indica* colonized the roots of inoculated plants and chlamydospores (piriform shaped) and mycelia (in some samples) were seen (Fig. 2 A) while no such spores and mycelia were observed in un-inoculated plants (Fig. 2 B). Thus, staining techniques and microscopic analysis confirmed the positive interaction (symbiotic association) of *P*. *indica* with inoculated plants.

### 3.3 Effect of As on osmotic tolerance indices

Electrolyte leakage was observed 13.8 μS/cm (2.8 fold increment) in un-inoculated plants at 150 μmol/L stress as compared to their respective control (4.9 μS/cm). However, electrolyte leakage was decreased by *P*. *indica* in inoculated plants as it was observed 10.2 μS/cm in *P*. *indica*-inoculated plants supplemented with 150 μmol/L stress. Likewise, significant difference was found under 50 and 100 μmol/L As stress. Similarly, relative water content of the inoculated plants was also higher as compared to un-inoculated plants. Relative water content was 65% higher in plants inoculated with *P*. *indica* and treated with 150 μmol/L stress in comparison with un-inoculated plants with the same As concentration (Fig. S2, A-B).

### 3.4 As concentration in roots and shoots

**Fig. 3:**
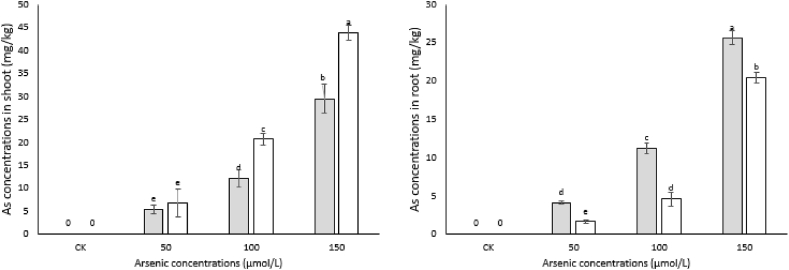
Arsenic concentrations (mg/kg) in the shoot (A) and root (B) of experimental Artemisia plants. Artemisia plants were co**-**inoculated with *P*. *indica* and supplemented with different arsenic concentrations or remained un-inoculated and treated with the same arsenic concentrations. The data are the mean values of three biological replicates with ±standard error. The same lower-case letters within each column indicates no significant difference among treatments (p < 0.05).

No As was found in the plants grown on uncontaminated substrate. As was mainly restricted and accumulated in the roots by *P*. *indica* in colonized plants. As concentration in the roots of inoculated plants increased (25.6 mg/kg) at 150 μmol/L stress while 20.4 mg/kg was the concentration in un-inoculated plants under the same (150 μmol/L) As stress (Fig. 3 B). Similarly, significant difference was observed in both treated and untreated plants with other stress levels i.e. 50 and 100 μmol/L. Remarkably, more As was mobilized in to the shoot of un-inoculated plants as compared to inoculated ones (Fig. 3 A). For example, 43.7 and 29.4 mg/kg of As was determined in the shoots of un-inoculated and inoculated plants, respectively under 150 μmol/L stress. Likewise, As concentration in the shoots was 6.4, 20.6, 5.4 and 12.1 mg/kg at 50 and 100 μmol/L stress, respectively, in un-inoculated and inoculated plants.

### 3.5 Regulation of antioxidant enzymes by *P*. *indica*

**Fig. 4:**
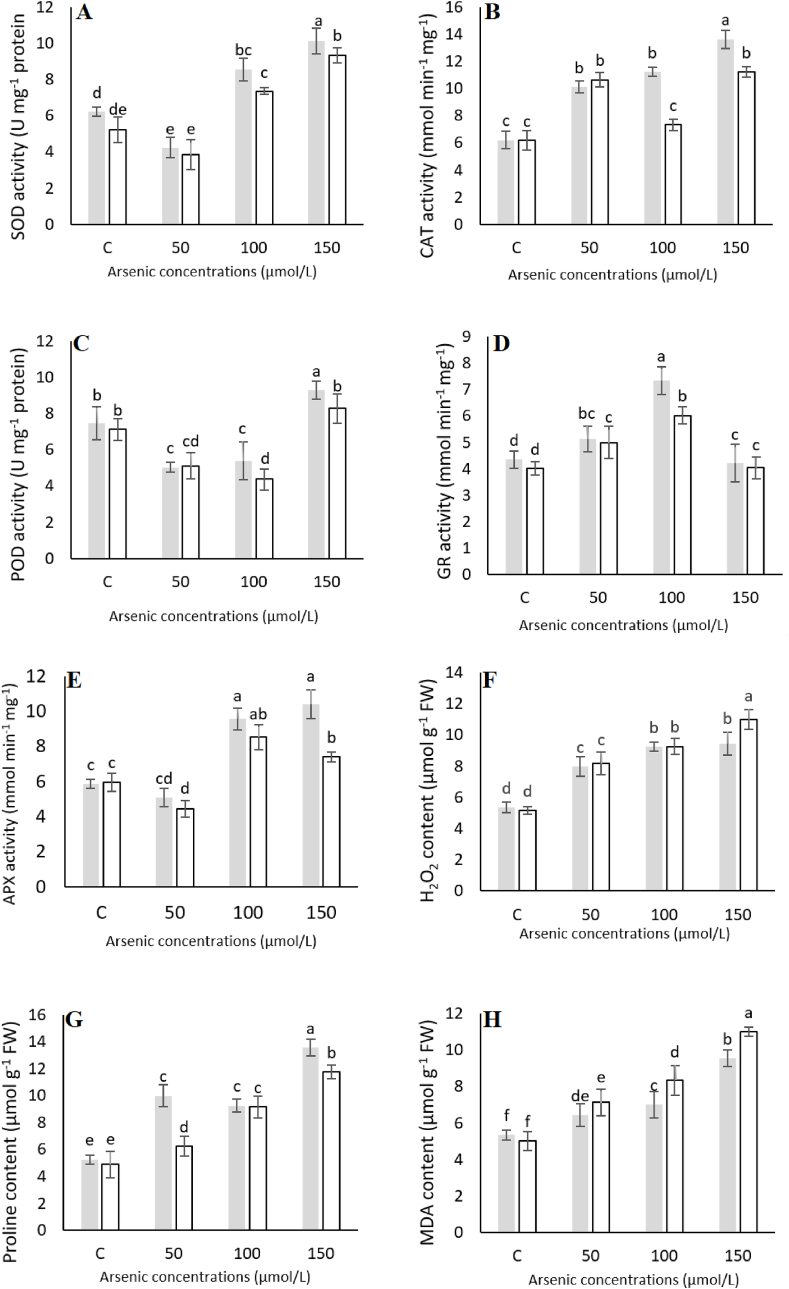
The enzymes activity (A–E) and H2O2 (F), Proline (G) and MDA content (H) in the leaf of As-treated Artemisia annua plants. Bars represent SEs, based on 3 independent experiments. Artemisia plants were co-inoculated with P. indica and supplemented with different arsenic concentrations or remained un-inoculated and treated with the same arsenic concentrations. The data are the mean values of three biological replicates with ±standard error. The same lower-case letters within each column indicates no significant difference among treatments (p < 0.05).

Overall, the activity of SOD was found to be increased under all concentrations of As stress (Fig. 4A–E). However, it was higher in control plants as compared to 50 μmol/L while in case of 100 and 150 μmol/L, the activity was 1.3 and 1.6 fold higher, respectively than that of control. Similarly, CAT activity also tends to be higher in inoculated plants except under 50 μmol/L, where it was slightly higher in un-inoculated plants. However, no significant difference was observed in positive (inoculated and without As) and negative (un-inoculated with As) control. The activity was significantly increased at 100 (1.5 fold) and 150 μmol/L (1.2 fold) in the colonized plants as compared to un-colonized plants under the same As concentrations. Under the same conditions, activity of POD also increased significantly under 150 μmol/L as compared to control, however, it decreased in case of 50 and 100 μmol/L comparatively to both positive and negative control (Fig. 4A–E). By contrast, GR activity was slightly increased in control plants in comparison with 150 μmol/L while under 50 and 100 μmol/L it was higher than that of control and inoculated and treated with 150 μmol/L As. Overall, however the activity was found to be increased in inoculated plants under all concentrations. The activity of APX was significantly higher in plants treated with 100 and 150 μmol/L stress than that of control while in case of 50 μmol/L the activity of control was observed higher. H_2_O_2_ content was observed significantly higher in As treated plants only at 150 μmol/L as compared to inoculated ones (Fig. 4F–H). However, no significant difference was observed in inoculated plants supplemented with 50 and 100 μmol/L As. Thus, *P*. *indica* significantly decreased H_2_O_2_ in colonized plants under 150 μmol/L As stress. Similarly, As treated plants showed significantly increased proline content than that of control. Plants colonized with *P*. *indica* also enhanced proline content significantly as compared to As alone under 50 and 150 μmol/L stress while no significant difference was observed under 100 μmol/L stress. Likewise, MDA content was also found to be increased in un-inoculated plants treated with As as compared to control. *P*. *indica* also significantly increased MDA content in inoculated plants than that of control (Fig. 4F–H).

### 3.6 Effect of *P*. *indica* on the level of phenolic acid, phenolic compound and flavonoid

**Fig. 5:**
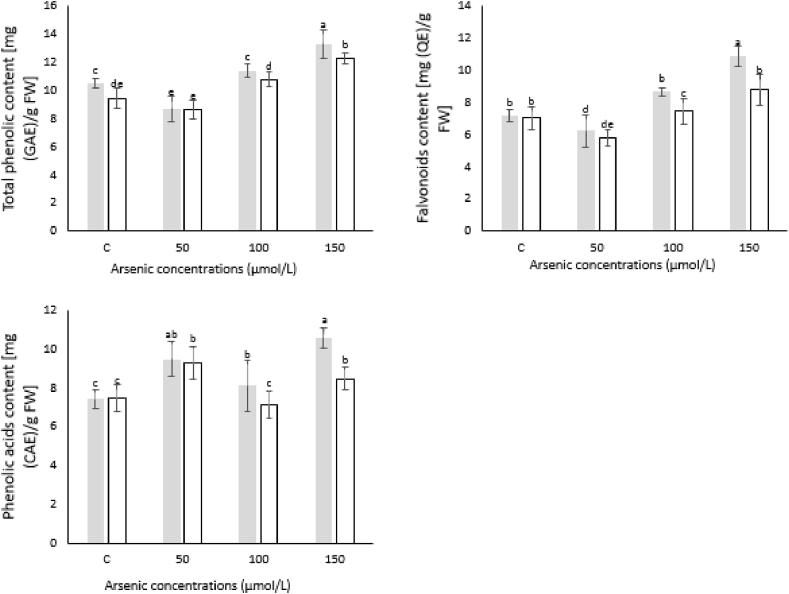
Effect of *P*. *indica* on total phenolic compounds (A), flavonoids content (B) and phenolic acids (C) of Artemisia leaves. Artemisia plants were co**-**inoculated with *P*. *indica* and supplemented with different arsenic concentrations or remained un-inoculated and treated with the same arsenic concentrations. The data is the mean values of three biological replicates with ±standard error. The same lower-case letters within each column indicates no significant difference among treatments (p < 0.05).

Phenolic acids content was significantly increased in *P*. *indica* colonized plants as compared to control. Similarly, higher phenolic acids were detected in inoculated plants treated with 150 μmol/L As stress followed by 50 μmol/L while plants treated with 100 μmol/L showed slightly higher content of phenolic acids as compared to control (Fig. 5). No significant difference was observed in positive and negative control plants. Likewise, significant increase was observed in total phenolic compounds content under 100 and 150 μmol/L As stress in inoculated plants as compared to control. However, less phenolic compounds were detected in both inoculated and un-inoculated plants treated with 50 μmol/L As stress than that of control. Besides, no significant difference was observed in inoculated and un-inoculated samples treated with 50 μmol/L As stress. Under similar conditions, flavonoids' production was increased in all given treatments except 50 μmol/L than that of control. The increment was 65.8% in inoculated plants treated with 150 μmol/L (Fig. 5).

### *3.7* *A. Annua* genes expression analysis through q-PCR

**Fig. 6:**
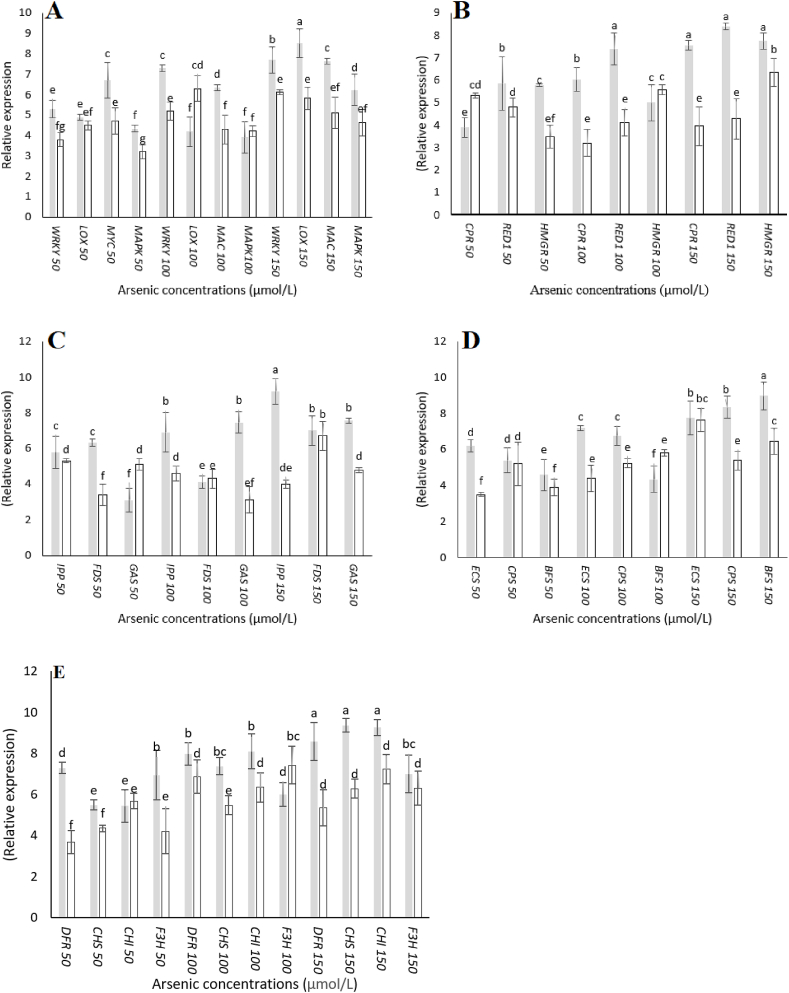
Relative expression level of selected genes in *P. indica* colonized and un-colonized *Artemisia annua* plants after AS treatment. Artemisia plants were co**-**inoculated with *P*. *indica* and supplemented with different arsenic concentrations or remained un-inoculated and treated with the same arsenic concentrations. The data is the mean values of three biological replicates with ±standard error. The same lower-case letters within each column indicates no significant difference among treatments (p < 0.05).

The transcriptional analysis of artemisinin biosynthetic pathway such as *RED1*, *CYP71AV1*, *DBR2*, *ADS* and *CRP*, flavonoids pathway (*F3H*, *CHS*, *CHI* and *FDR*), isoprenoid pathway (*FDS*, *HMGR*, *IPP*), terpenes biosynthesis pathway (*BFS*, *GAS*, *CPS*, *ECS*) and important signaling process (*MYC*, *LOX*, *WRKY*, *MAPK*) genes were analyzed in both inoculated and un-inoculated As treated plants (Fig. 6A–E). Although genes expression was upregulated in inoculated plants treated with 50 and 100 μmol/L As but the expression was found to be higher (in most if not all) inoculated plants treated with 150 μmol/L As. Similarly, the expression pattern of *MYC*, *LOX*, *WRKY*, *MAPK* (regulatory genes mostly involved in signaling process) was also upregulated in inoculated plants treated with 150 μmol/L As. These, results revealed that inoculated and treated with 150 μmol/L As treatment was more effective than inoculated 50 and 100 μmol/L As.

### 3.8 HPLC analysis of artemisinin and flavonoids content

The transcript levels of the studied genes were increased in most if not all of inoculated plants treated with different concentrations of arsenic as compared to control and un-inoculated but treated plants as reveled by real-time PCR analysis. Considering these results, HPLC analysis was carried out in order to measure the concentration of artemisinin content in both inoculated and un-inoculated plants. A significant increase was found in artemisinin content (as expected from relative expression study of artemisinin biosynthetic pathway genes) in inoculated and treated with As (except 50 μmol/L) as compared to control and un-inoculated treated plants. Artemisinin concentration was increased 1.3 and 1.4 fold in inoculated and treated with 100 and 150 μmol/L As, respectively, as compared to positive control while artemisinin concentration was decreased 0.9 fold in inoculated and treated with 50 μmol/L As stress as compared to control (Fig. S3, A-B). Similarly, genes' expression (analyzed through q-PCR) of flavonoids biosynthetic pathway was also found to be increased in inoculated plants treated with different concentrations of As. Therefore, HPLC analysis was also performed in order to measure flavonoids content. A total of 9 flavonoids were used as standard to compare and confirm the detected flavonoids and their levels. Out of 9, 6 flavonoids (Syringic acid, Ferulic acid, Luteolin, Chlorogenic acid, Rutin trihydrate and Kaempferol) were increased significantly in inoculated treated with 50 μmol/L arsenic as compared to control while Quercetin and Gallic acid were decreased and Hydroxycinnamic acid was not detected (Table 1). Under similar conditions, Syringic acid, Ferulic acid, Luteolin, Chlorogenic acid, Quercetin, Rutin trihydrate, Kaempferol, and Hydroxycinnamic acid were found increased, 1.5, 1.3, 3.5, 1.2, 1.4, 2.4, 2.9 and 0.5 fold, respectively in inoculated and treated with 100 μmol/L arsenic as compared to un-inoculated plants. Likewise, these compounds were increased significantly except Rutin trihydrate and Kaempferol in inoculated plants treated with 150 μmol/L As.

**Table 1:** Quantitative analysis of phenolic compounds in the leaves of *Artemisia annua* after treatments.

Treatments	Syringic acid	Ferulic acid	Luteolin	Chlorogenic acid	Quercetin	Rutin trihydrate	Kaempferol	Hydroxycinnamic acids	Gallic acid
0 mg/kg									
***P. indica***	2.39 ± 0.135c	3.04 ± 0.27d	0.27 ± 0.03d	8.71 ± 0.81e	3.87 ± 0.10c	7.29 ± 4.77bc	2.38 ± 0.34b	2.3 ± 1.38b	2.34 ± 1.35ab
**Un-inoculated**	1.44 ± 0.012d	1.36 ± 0.07e	0.20 ± 0.02d	3.64 ± 2.01e	1.17 ± 0.11d	1.77 ± 0.65e	8.43 ± 5.98a	8.43 ± 5.9a	6.43 ± 5.56ab
**50 mg/kg**									
***P. indica***	4.73 ± 0.63b	8.77 ± 0.65a	0.45 ± 0.75c	134.2 ± 29.8c	0.62 ± 3.38a	9.26 ± 0.14b	3.30 ± 0.20a	ND	1.59 ± 0.05b
**Un-inoculated**	1.50 ± 0.23d	5.71 ± 0.56b	0.18 ± 0.03d	106.8 ± 2.64d	3.99 ± 0.51c	4.42 ± 0.50de	1.13 ± 0.02c	0.31 ± 0.01b	3.99 ± 5.37ab
**100 mg/kg**									
***P. indica***	6.71 ± 0.92a	7.86 ± 0.58a	1.30 ± 0.62c	163.7 ± 9.93b	7.03 ± 0.76ab	15.88 ± 2.03a	3.27 ± 0.17a	0.50 ± 0.03b	2.37 ± 0.11ab
**Un-inoculated**	4.41 ± 0.81b	5.80 ± 0.60b	0.37 ± 0.07c	134.3 ± 15.8c	4.73 ± 0.02bc	5.58 ± 2.45cd	1.12 ± 0.07c	0.23 ± 0.09b	7.14 ± 8.07ab
**150 mg/kg**									
***P. indica***	2.59 ± 0.31c	5.06 ± 1.44b	7.38 ± 0.99a	229.4 ± 3.43a	5.23 ± 0.61b	9.19 ± 0.92bc	1.11 ± 0.01c	0.26 ± 0.014b	11.51 ± 7.66a
**Un-inoculated**	1.59 ± 0.25d	4.41 ± 0.55c	4.43 ± 0.87b	225.3 ± 7.84a	3.20 ± 0.55dc	9.72 ± 0.02b	2.09 ± 0.01b	0.28 ± 0.02b	2.32 ± 0.05ab

Artemisia plants were co**-**inoculated with *P*. *indica* and supplemented with different arsenic concentrations or remained un-inoculated and treated with the same arsenic concentrations. The data is the mean values of three biological replicates with ±standard error. In the table, ND stands for not detected.

## 4 Discussion

Except a few plant species of the family Juncaceae, Amaranthaceae, Proteaceae, Brassicaceae, Cyperaceae and Chenopodiaceae, terrestrial plants (almost all) make symbiotic relationship with arbuscular mycorrhizal fungi (AMF) in the natural habitat, resulting in a number of benefits to their hosts. These fungi promote nutritional status of the plant and confers resistance to a number of biotic and abiotic stresses including drought, salt and heavy metals (Kumar et al., 2009, 2011; Orłowska et al., 2012; Spagnoletti and Lavado, 2015). However, the beneficial consequences of *P*. *indica* are much more than that of AMF (Varma et al., 1999; Yadav et al., 2010; Jogawat et al., 2013). In this research work, the results showed that *P*. *indica* colonized plants' root grown in substrate experimentally contaminated with different concentrations of As even at 150 μmol/L, suggesting that the fungus is capable of making symbiotic association under high As stress. Notably, *P*. *indica* is able to enhance plant growth and development (Gill et al., 2016; Mou, 2017; Dehury et al., 2018). Additionally, the beneficial effect of *P*. *indica* under abiotic stresses such that drought stress in barely (Ghaffari et al., 2019), Arabidopsis (Sherameti et al., 2008) and *Zea mays* (Hosseini et al., 2019); and salt stress in tomato (Ghorbani et al., 2019) and melon (Hassani et al., 2019) have been previously reported. Likely, plant growth was promoted in *P*. *indica* colonized stressed Artemisia plants than that of un-inoculated stressed plants in our study (Fig. S4, A-B). Like that, adverse effect of heavy metal (Cd) was overcome in tobacco by *P*. *indica* (Hui et al., 2015). According to (Shahabivand et al., 2012) Cd lower fresh weight and reduced wheat growth while *P*. *indica* ameliorated detrimental effects and promoted growth in colonized stressed plants as compared to their respective control. *P*. *indica* could enhance plant growth by taking more nutrients (Hartley and Gange, 2009), consequently helps to activate different pathways (particularly biochemical) required to achieve proper growth (Vadassery et al., 2009). The most significant characteristic has been thought to be the restriction of heavy metals in roots and low transportation to the shoot which determines storage, translocation and metal tolerance in the plants (Dickinson and Lepp, 1997). We have also shown that *P*. *indica* accumulated and restricted As into roots and translocation is reduced to the shoots where the results are in accordance with the previous research (Mohd et al., 2017). Immobilization process of metal in the roots is mostly due to compartmentalization, adsorption and transferring metals into precipitates (Mohd et al., 2017). Osmotic stress was significantly alleviated by *P*. *indica* in rice and further confers resistance against drought stress. Like that, in our study, *P*. *indica* mediated reduction in As stress (as revealed by attenuated ROS generation) despite of high As levels, indicates fungus tolerance to heavy metalloids. Also, the susceptibility of colonized plants to As was significantly lowered by *P*. *indica*. Other researchers, for example (Shahabivand et al., 2017; Nanda and Agrawal, 2018), have also been shown that *P*. *indica* is able to immobilize different heavy metalloids including As into roots and reduced their translocation to the aerial plant parts. Importantly, in symbiotic association plants mainly restrict metals in roots (by binding it to hyphal cell wall) (Emamverdian et al., 2015). Furthermore, different functional groups in the fungal cell walls could help in metal tolerance by binding these ions during the biosorption process (Xu et al., 2012). *Exophiala pisciphila* (a metal tolerant fungus) has successfully accumulated more than 5% Cd of its dry weight (intracellularly) (Zhang et al., 2008) which enhances plant's tolerance to Zn, Pb and Cd (Li et al., 2011). Thus, it is suggested that by immobilizing As in roots, *P*. *indica* can confers As tolerance (as showed by *in vitro* assays in our study), also indicating fungus detoxification capacity and may provide a best strategy to restrict heavy metals in polluted areas. Additionally, enzymatic activities were significantly enhanced by *P*. *indica*, resulting in improved antioxidant defense system. Rapid conversion of superoxide into H_2_O_2_ by SOD in mitochondria, apoplasts, nuclei, chloroplasts and peroxisomes have been reported earlier (Gill and Tuteja, 2010). Increment in SOD activity in *P*. *indica* colonized plants was observed in our study where similar results i.e. incensement of SOD activity in *P*. *indica* inoculated plants but under different metal stress have also been reported by (Hui et al., 2015). It has also been suggested that plants' antioxidant enzyme system is activated by fungus (Baltruschat et al., 2008) which indicates that *P*. *indica* (in our study) might have used the same mechanism(s) for activation of antioxidant enzymes to prevent ROS induced oxidative damage. In addition to improving other important protective parameters, the fungus also successfully lowered down H_2_O_2_ and MDA content which are thought to be damaging factors. Similarly, GR activity was significantly increased in *P*. *indica* colonized plants in our study which has a significant role in maintaining GSH/GSSG ratio, resulting in high maintenance of GSH pool (which is important for proper protein function) under normal and detrimental conditions (Balestrini et al., 2012). Therefore, increase in GR activity in inoculated plants might be responsible for enhanced redox balance, resulting in ROS reduction and oxidative damage (Balestrini et al., 2012). A significant increase was also found in proline content in colonized treated plants (except those colonized and treated with 100 μmol/L As). It has been reported previously that fungi induce proline accumulation (Jogawat et al., 2013) which in turn assist to overcome the detrimental effects of ROS (Spagnoletti and Lavado, 2015).

Flavonoids are important secondary metabolites acts as ROS scavenging system and their role in mitigation of various abiotic stresses have been reported (Martinez et al., 2016). Like flavonoids, being important secondary metabolites, artemisinin have also been reported with diverse functions. Both flavonoids and artemisinin contents were significantly increased in inoculated plants treated with As. Our findings suggest that As tolerance of Artemisia was mainly associated with the increased level of As immobilization/accumulation in the fungus inoculated plant roots. The major mechanism could be As accumulation by fungus hyphal mass and spores within the root tissues that further restricted As accumulation in microbial cells and thus preventing its transportation to the shoots. Thus, *P*. *indica* can be highly recommended as a best candidate for phytostabilization to combat and or reduce adverse effects of heavy metals on plants growing in heavy metals contaminated environments.

## 5 Conclusion and future prospects

Adverse effects of heavy metals have severe and sometimes lethal effects on living organisms including plants and human beings. Due to certain characteristics such as non-biodegradability, heavy metal(loid)s can persist for long period in the soil. Rectification/biotransformation of soils contaminated with heavy metals and/or metalloids (Cu, Pb, As, Cd etc.) could be possible through fungi due to their physiology (production of important secondary metabolites, enzymes and high degree of tolerance to biotic and abiotic stresses) and mycelial-morphology (highly extensive and reactive biological surface). Furthermore, fungi can effectually biotransform and/or biosequestrate many contaminants (both organic and inorganic) into non-bioavailable form (Albert et al., 2018). In this experiment, *P*. *indica* showed high degree of tolerance to As stress *in vitro* (both on agar/solid and liquid broth media). Also, the bioaccumulation potential of *P*. *indica* was confirmed as it restricted and accumulated most of the As in the roots of inoculated plants while a negligible amount was translocated to the shoots. Other important parameters such as biochemical, physiological, morphological and molecular also showed reasonable variation in *P*. *indica*-inoculated plants. Therefore, *P*. *indica* and perhaps other endophytes could be used to rectify heavy metal(loid)s contaminated soils. However, studies are required to find the molecular mechanism(s) of *P*. *indica*-host plants' interaction and biosorption/tolerance profiles with other heavy metal(loid)s.

## Author's contribution statement

**Saeed-ur-Rahman:** participated in designing of the study and carried out the experimental work, read and approved the final manuscript. **Muhammad Khalid:** participated in designing of the study and carried out the experimental work, read and approved the final manuscript. **Sadaf-Ilyas Kayani:** interpreted the data and drafted the manuscript, read and approved the final manuscript. **Kexuan Tang:** interpreted the data and drafted the manuscript, provided experimental resources and participated in data analysis as well as drafting the manuscript, read and approved the final manuscript, All the research work was carried out under the supervision of (Principal Investigators of the project) who designed and coordinated the experiments.

## Funding information

This research was supported by grants from the Bill & Malinda 10.13039/100000865Gates Foundation (OPP1199872), the China 10.13039/501100012166National Key Research and Development Program (2017ZX09101002-003-002), and the Young Scientists Fund of the 10.13039/501100001809National Natural Science Foundation of China (Grant No. 31600231).

## Declaration of competing interest

The authors declare that they have no known competing financial interests or personal relationships that could have appeared to influence the work reported in this paper.
