# Supplementary material for: The ameliorative effects of exogenous inoculation of Piriformospora indica on molecular, biochemical and physiological parameters of Artemisia annua L. under arsenic stress condition
Source: Ecotoxicol Environ Saf. 2020 Dec 15;206:111202. doi: 10.1016/j.ecoenv.2020.111202 (PMC7646201; doi:10.1016/j.ecoenv.2020.111202)
Supplement: Multimedia component 1 [file mmc1.docx]

**Supplementary Material**

**The ameliorative effects of exogenous inoculation of *Piriformospora indica* on molecular, biochemical and physiological parameters of *Artemisia annua* L. under arsenic stress condition**

**Saeed-ur-Rahman^1^, Muhammad Khalid^2^, Sadaf-Ilyas Kayani^1^ and Kexuan Tang**^*^**^1^**

^1^Joint International Research Laboratory of Metabolic & Developmental Sciences, Key Laboratory of Urban Agriculture (South) Ministry of Agriculture, Plant Biotechnology Research Center, Fudan-SJTU-Nottingham Plant Biotechnology R&D Center, School of Agriculture and Biology, Shanghai Jiao Tong University, Shanghai 200240, China

^2^Key Laboratory of Urban Agriculture, School of Agriculture and Biology, Shanghai Jiao Tong University, Shanghai, China

^*^Corresponding author:

Email address: kxtang@sjtu.edu.cn


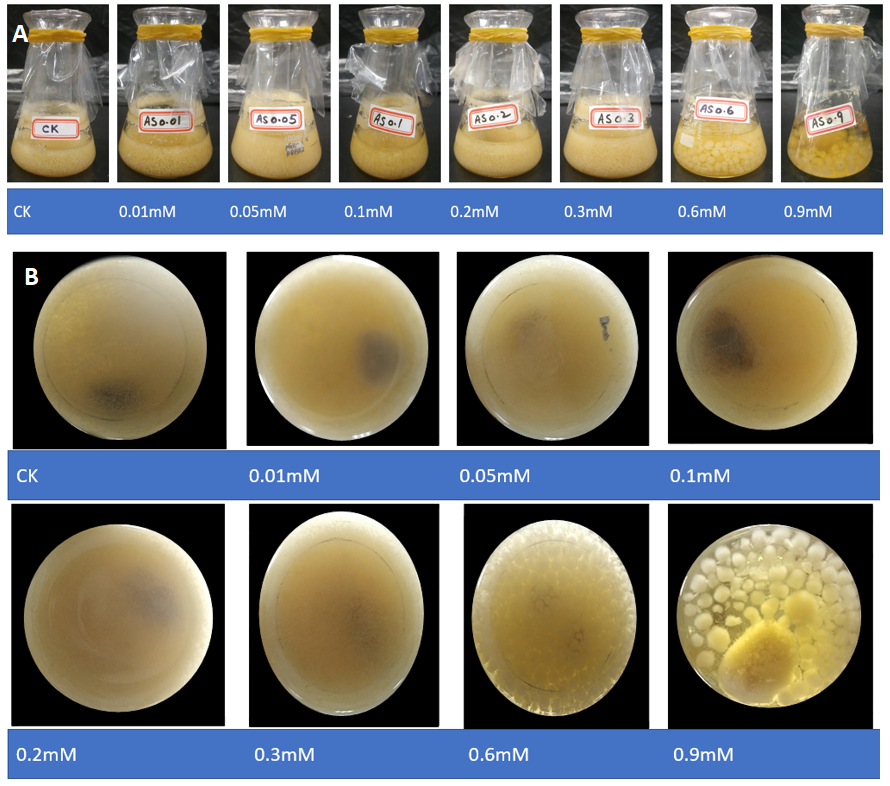


Fig S1. The response of *P*. *indica* growth (A-B) to different concentrations of arsenic (µmol/L) in the liquid culture, 15 days after fungus inoculation.


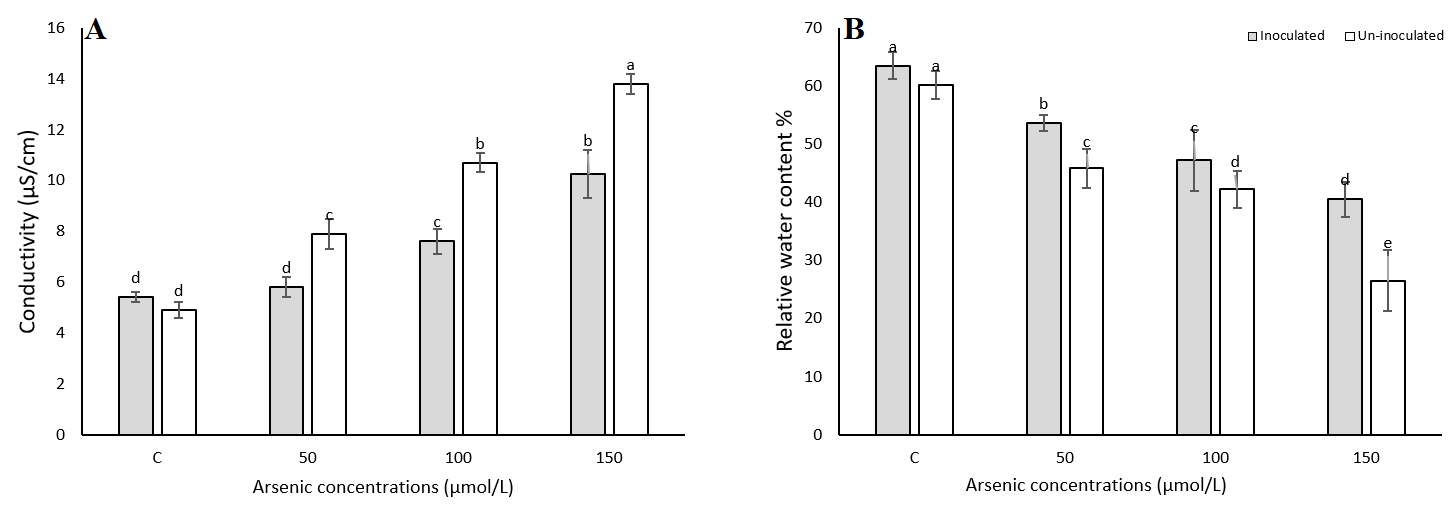


Fig S2. Effects of arsenic stress on electrolyte leakage (A) and relative water content (%) (B). Artemisia plants were co**-**inoculated with *P*. *indica* and supplemented with different arsenic concentrations or remained un-inoculated and treated with the same arsenic concentrations. The data is the mean values of three biological replicates with ± standard error. The same lower-case letters within each column indicates no significant difference among treatments (p < 0.05).


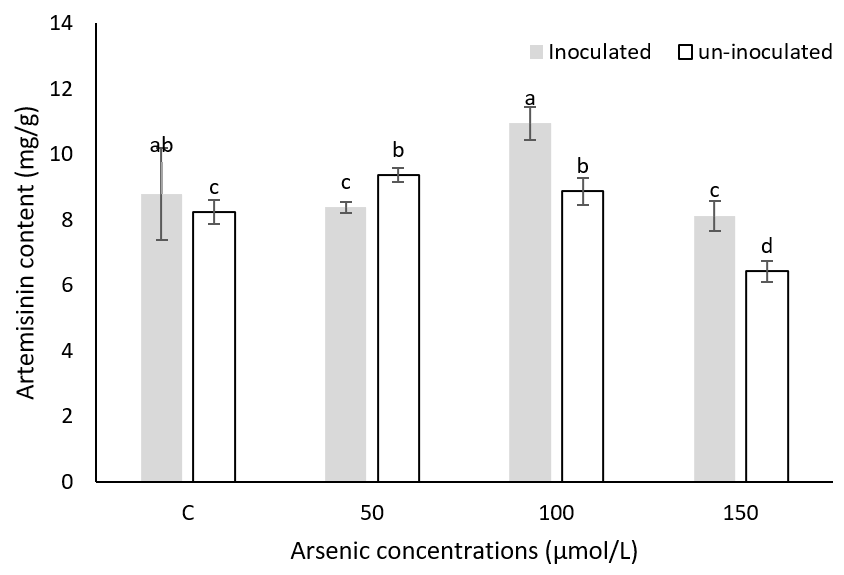


Fig S3. Artemisinin content in the leaf of AS-treated *Artemisia annua* plants. Bars represent SEs, based on 3 independent experiments. Artemisia plants were co**-**inoculated with *P*. *indica* and supplemented with different arsenic concentrations or remained un-inoculated and treated with the same arsenic concentrations. The data is the mean values of three biological replicates with ± standard error. The same lower-case letters within each column indicates no significant difference among treatments (p < 0.05).

**
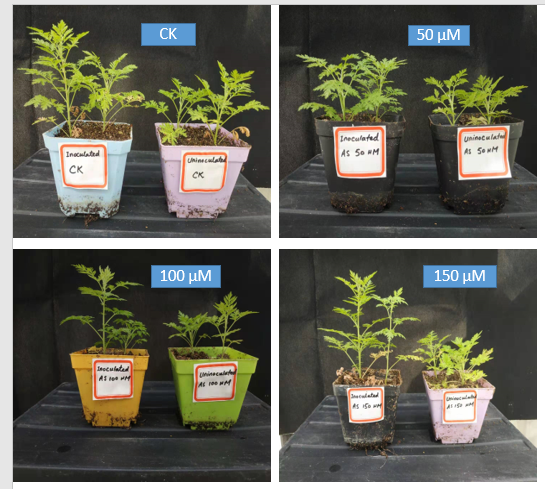
**

Fig S4. Plants morphology under given treatments. Artemisia plants were co**-**inoculated with *P*. *indica* and supplemented with different arsenic concentrations or remained un-inoculated and treated with the same arsenic concentrations.
